# Perfil farmacoterapêutico de pacientes obesos no pós-operatório de cirurgia bariátrica

**DOI:** 10.1590/1677-5449.002016

**Published:** 2016

**Authors:** Elenara Simoni Kovaleski, Helena Schroeder, Mauricio Krause, Caroline Dani, Patrícia Martins Bock

**Affiliations:** 1 Centro Universitário Metodista IPA, Porto Alegre, RS, Brasil.; 2 Universidade Federal de Ciências da Saúde de Porto Alegre – UFCSPA, Porto Alegre, RS, Brasil.; 3 Universidade Federal do Rio Grande do Sul – UFRGS, Porto Alegre, RS, Brasil.

**Keywords:** obesidade, cirurgia bariátrica, atenção farmacêutica, farmacoterapia

## Abstract

**Contexto:**

A obesidade pode estar relacionada a doenças como diabetes, hipertensão arterial e dislipidemia. A cirurgia bariátrica é um dos tratamentos mais eficazes, levando à diminuição de peso e comorbidades.

**Objetivo:**

Avaliar o perfil metabólico e farmacoterapêutico de pacientes obesos após cirurgia bariátrica.

**Métodos:**

Trata-se de um estudo observacional transversal retrospectivo, realizado em um hospital localizado na cidade de Porto Alegre, RS, Brasil. Foram avaliados 70 prontuários de pacientes que realizaram cirurgia bariátrica, nos períodos de antes de 2 meses e mais de 6 meses após a cirurgia bariátrica. A análise estatística foi realizada no programa SPSS 17.0®.

**Resultados:**

A pressão arterial inicial foi de 130/85 mmHg, passando para 120/80 mmHg (p < 0,01). Com relação ao perfil metabólico antes de dois meses, o HDL foi de 34 mg/dL, o colesterol total foi de 195,07 ± 40,17 mg/dL, o LDL foi de 118,22 ± 41,28 mg/dL, os triglicerídeos foram de 141,09 ± 43,39 mg/dL, e a glicemia de jejum foi de 90 mg/dL. Após 6 meses de cirurgia, os valores passaram para 43 mg/dL, 133,67 ± 28,14 mg/dL, 65,53 ± 24,3 mg/dL, 104,41 ± 29,6 mg/dL, e 77 mg/dL, respectivamente (p < 0,01). Com relação ao uso de medicamentos, 41% utilizaram anti-hipertensivos, 39% utilizaram hipolipemiantes, 10% utilizaram hipoglicemiantes orais e 97% utilizaram suplementos antes dos 2 meses de cirurgia. Após os 6 meses, os percentuais foram alterados para 21%, 19%, 9% e 99%, respectivamente.

**Conclusões:**

O estudo mostra o sucesso da cirurgia bariátrica em pacientes obesos com comorbidades, revelando melhora no perfil metabólico e redução na utilização de medicamentos para tratamento de comorbidades.

## INTRODUÇÃO

A obesidade é uma doença crônica e multifatorial que está relacionada a fatores nutricionais, genéticos, culturais, psicossociais e comportamentais^1^. Em todo o mundo, pelo menos 2,8 milhões de pessoas morrem por ano como resultado da obesidade e seus agravos. Nas Américas, é encontrada a maior prevalência de sobrepeso e obesidade (62% e 26%, respectivamente) para ambos os sexos^2^. Além disso, a obesidade está associada ao desenvolvimento de diversas doenças, como diabetes melito tipo 2 (DM2), hipertensão arterial, alguns tipos de câncer e doenças cardiovasculares^3^^,^ 4.

O diagnóstico é realizado por profissionais de saúde especializados, que fazem parte de uma equipe multidisciplinar que avalia o paciente e o encaminha ao tratamento mais adequado. Entre os tratamentos utilizados para controle da obesidade, incluem-se o uso de medicamentos, dietas de baixas calorias, atividades físicas, mudanças no estilo de vida, mudanças culturais e, finalmente, a cirurgia bariátrica. A cirurgia bariátrica é recomendada apenas para indivíduos que apresentam obesidade mórbida ou obesidade grave associada a comorbidades [índice de massa corporal (IMC) ≥ 40 kg/m^2^ ou ≥ 35 kg/m^2^ com comorbidades associadas]^5^^-^ 7. A cirurgia bariátrica é indicada para pacientes que passaram por outros tratamentos por no mínimo dois anos, como tratamentos farmacológicos, prática de atividade física, dietoterapia e psicoterapia, e tiveram insucesso nesses tratamentos. Dessa forma, a cirurgia é indicada como último recurso, porém é um dos tratamentos com maior eficácia. Ela consiste em uma intervenção cirúrgica realizada no estômago ou intestino para reduzir o volume das refeições ingeridas e aumentar os sinais de saciedade^8^.

Para o tratamento cirúrgico da obesidade mórbida, as cirurgias aceitas são as restritivas, disabsortivas ou mistas. Entre elas, a mais empregada é a mista predominantemente restritiva, que consiste na derivação gástrica em Y de Roux (DGYR), conhecida como Fobi-Capella, que oferece uma perda de peso consistente, uma boa tolerância da parte do paciente e uma taxa aceitável de complicações pós-cirúrgicas ao longo do tempo^9^^,^ 10.

Pacientes que se submetem à cirurgia apresentam diminuição das comorbidades associadas à obesidade, como IMC, peso, pressão arterial, glicemia, triglicerídeos, colesterol total e LDL (*low-density lipoprotein*), e aumento das frações de HDL (*high-density lipoprotein*)^11^. Mas, após a cirurgia, o paciente segue tratamento contínuo com medicamentos anti-hipertensivos, hipoglicemiantes e hipolipemiantes, e inicia o uso de suplementos^5^. O uso de suplementos, como vitamina B12, ferro, cálcio, entre outros, é indispensável no pós-operatório do paciente submetido a cirurgia bariátrica devido à diminuição da absorção desses nutrientes, ocorrida principalmente no intestino, pelo grau de restrição causado pela cirurgia^12^^,^ 13.

Este estudo teve como objetivo avaliar o perfil farmacoterapêutico dos pacientes obesos de um hospital no sul do Brasil, nos períodos de menos de 2 meses e após 6 meses da realização da cirurgia bariátrica, uma vez que esse perfil pode ser alterado em resposta aos parâmetros fisiopatológicos modificados pela cirurgia.

## MÉTODOS

Este trabalho é um estudo transversal retrospectivo com levantamento epidemiológico e análise de dados qualitativos e quantitativos. O objetivo foi avaliar o perfil farmacoterapêutico de pacientes obesos que fazem uso de medicamentos para o tratamento de doenças associadas à obesidade e suplementos no período de menos de 2 meses e mais de 6 meses após a cirurgia bariátrica. O trabalho foi desenvolvido nas dependências de um hospital de grande porte localizado na cidade de Porto Alegre, RS, Brasil. Trata-se de um hospital privado que atende pacientes particulares ou convênios. Foram avaliados 70 prontuários de pacientes que realizaram cirurgia bariátrica, pertenciam ao Grupo Novo Peso e preencheram os critérios de inclusão, no período de janeiro de 2010 a janeiro de 2012.

Os prontuários inclusos na pesquisa foram aqueles de pacientes que haviam realizado cirurgia bariátrica e apresentaram informações completas para a análise de dados farmacológicos, clínicos, demográficos e antropométricos. Foram excluídos os prontuários dos pacientes que não apresentaram informações completas para a análise de dados e dos pacientes que realizaram a cirurgia, mas não retornaram para acompanhamento através do Grupo Novo Peso.

As variáveis pesquisadas foram: idade; gênero; estado civil; histórico de obesidade familiar, diabetes, problemas cardíacos, hipertensão arterial; prevalência de diabetes, problemas cardíacos e hipertensão arterial; peso, IMC, circunferência da cintura (CC), pressão arterial, dados laboratoriais (glicemia, triglicerídeos, colesterol total e HDL); e todos os medicamentos utilizados para o tratamento de doenças associadas à obesidade em pacientes obesos no período de até 2 meses e mais de 6 meses após a cirurgia bariátrica.

A coleta de dados foi iniciada somente após a aprovação do projeto pelo Comitê de Ética em Pesquisa do Centro Universitário Metodista IPA, pelo protocolo nº 213/2012. A privacidade dos pacientes foi mantida sem causar nenhum prejuízo a eles.

Os dados levantados foram analisados pelo programa Statistical Package for the Social Sciences (SPSS) versão 17.0®. A análise descritiva foi realizada através da apresentação dos resultados em frequências (variáveis qualitativas), média e desvio padrão para as variáveis que apresentaram distribuição normal, ou mediana e intervalo interquartis (variáveis quantitativas). Para comparação dos parâmetros antes de 2 meses e após 6 meses, foi utilizado o teste *t* para as variáveis que apresentaram distribuição normal, e para as que não apresentaram distribuição normal foi realizado o teste de Mann-Whitney.

## RESULTADOS

Foram analisados 70 prontuários de pacientes no período de menos de 2 meses e após 6 meses da cirurgia bariátrica. A Tabela 1 apresenta o perfil dos pacientes em estudo. Quanto à distribuição dos fatores de risco dos familiares dos pacientes, 75,7% (n = 53) apresentaram histórico de obesidade, 42,9% (n = 30), de diabetes, 54,3% (n = 38), de problemas cardíacos, e 70% (n = 49), de hipertensão arterial.

**Tabela 1:** Perfil dos pacientes.

**Características gerais**		
**Variáveis**	**Frequência absoluta (n)**	**Frequência relativa (%)**
Gênero		
Feminino	58	82,9
Masculino	12	17,1
Idade		
20-29 anos	15	21,4
30-39 anos	29	41,4
40-49 anos	15	21,4
50-59 anos	10	14,3
60-69 anos	1	1,4
Estado civil		
Solteiro	28	40,0
Casado	33	47,1
Divorciado	9	12,9
Histórico de obesidade		
Sim	53	75,7
Não	17	24,3
Histórico de diabetes		
Sim	30	42,9
Não	40	57,1
Histórico de problemas cardíacos		
Sim	38	54,3
Não	32	45,7
Histórico de hipertensão arterial		
Sim	49	70,0
Não	21	30,0

**Figura 1:**
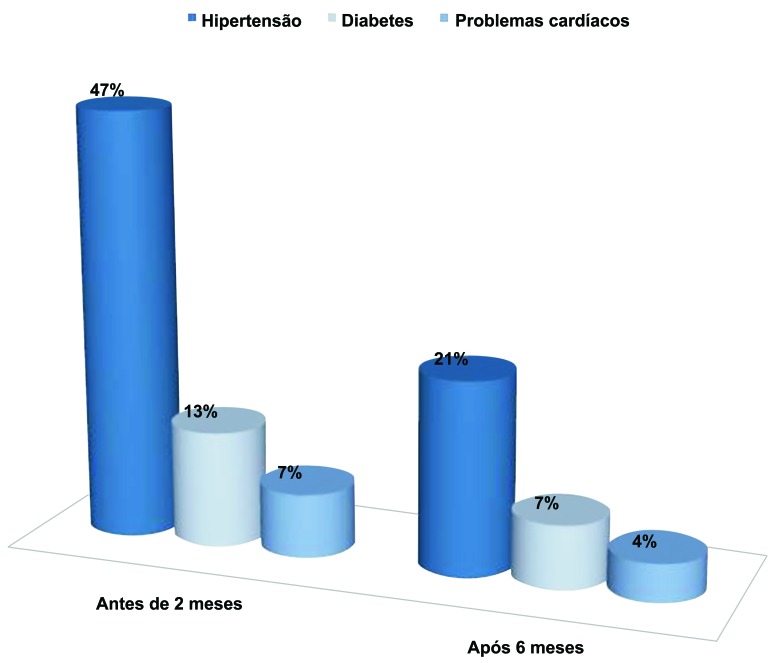
Doenças associadas à obesidade.

A Figura 1 mostra a frequência de comorbidades verificada nos pacientes. Observou-se que 47% (n = 33) apresentaram hipertensão arterial, 12% (n = 9) apresentaram DM2 e 7% (n = 5) apresentaram problemas cardíacos antes de 2 meses da cirurgia bariátrica. Passados 6 meses da cirurgia, apenas 36% (n = 25) dos pacientes apresentaram hipertensão arterial, 7% (n = 5) apresentaram DM2 e 7% (n = 5) apresentaram problemas cardíacos.

Com relação à prática de atividade física nessa população, o estudo mostra que somente 24% (n = 17) realizavam algum tipo de exercício antes de 2 meses de cirurgia, número que aumentou para 86% (n = 60) após 6 meses. A maioria da população praticava caminhadas como atividade regular.

A Tabela 2 mostra os resultados de pressão arterial, CC, IMC, HDL-C, glicemia, triglicerídeos e peso corporal. A pressão arterial sistólica apresentou mediana de 130 mmHg (120-150) antes de 2 meses e 120 mmHg (120-130) após 6 meses de cirurgia bariátrica. Já a pressão arterial diastólica apresentou mediana de 85 mmHg (80-90), que reduziu para 80 mmHg (80-80), valores significativos neste estudo (p < 0,01), conforme teste de Mann-Whitney.

**Tabela 2:** Alterações metabólicas.

**Variáveis**	**Antes de 2 meses**	**Após 6 meses**
Pressão arterial (mmHg)		
Sistólica	130 (120-150)	120 (120-130)^*^
Diastólica	85 (80-90)	80 (80-80)^*^
Circunferência da cintura (cm)	127 (118-133)	87 (83-99)^*^
Glicemia	90,50 (86-100)	77,50 (69-84)^*^
IMC	41,44 (39-44)	25 (23-28)^*^
HDL-C	34 (32-40)	43 (41-52)^*^
LDL	118,22 ± 41,28	62,53 ± 24,3^**^
CT	195,07 ± 40,17	133,67 ± 28,14^**^
Triglicerídeos	141,09 ± 43,39	104,41 ± 29,60^**^
Peso	113 ± 21,5	71,07 ± 14,69^**^

* Os valores são apresentados como mediana e intervalo interquartis com p ˂ 0,01, segundo teste de Mann-Whitney.

** Os valores são apresentados como média e desvio padrão com p ˂ 0,01, segundo teste *t*.

IMC = índice de massa corporal; HDL-C = lipoproteína de alta densidade; LDL = lipoproteína de baixa densidade; CT = colesterol total.

A medida da CC dos pacientes apresentou mediana de 127 cm (118-133) antes de 2 meses e 87 cm (83-99) após 6 meses (p < 0,01), valores significativos pelo teste de Mann-Whitney. O IMC apresentou uma mediana de 41 kg/m^2^ (39-44) antes de 2 meses, reduzindo consideravelmente para 25 kg/m^2^ (23-28) após 6 meses de cirurgia bariátrica, o que foi estatisticamente significativo neste estudo (p < 0,01), segundo teste de Mann-Whitney. O peso corporal apresentou média e desvio padrão de 113 ± 21,5 antes de 2 meses e 71,07 ± 14,69 após 6 meses. Dos 70 pacientes, 59% apresentavam peso acima de 105 kg antes de 2 meses de cirurgia bariátrica. Após 6 meses da cirurgia, 97% dos pacientes apresentaram peso abaixo de 105 kg, e 46% apresentaram peso entre 65 e 85 kg (p < 0,01).

HDL, colesterol total e LDL apresentaram níveis alterados antes de 2 meses de cirurgia. Verificou-se que o HDL apresentou mediana de 34 (32-40), o colesterol total apresentou média e desvio padrão de 195,07 ± 40,17, e o LDL apresentou média de 118,22 ± 41,28. Após 6 meses de cirurgia, os valores passaram para 43 (41-52), 133,67 ± 28,14 e 65,53 ± 24,3, respectivamente. Quanto aos triglicerídeos, a média e o desvio padrão foram de 141,09 ± 43,39 antes dos 2 meses, reduzindo para 104,41 ± 29,6 após 6 meses, valores significativos (p < 0,01), segundo teste *t*. Já a glicemia de jejum dos pacientes antes de 2 meses apresentou mediana de 90 mg/dL (86-100), reduzindo para 77 mg/dL (69-84) após 6 meses de cirurgia bariátrica, sendo esses valores significativos (p < 0,01), segundo teste de Mann-Whitney. Nos 2 meses após a cirurgia, 11 pacientes apresentaram glicemia de jejum alterada (≥ 100 mg/dL), sem uso de medicamentos.

**Figura 2:**
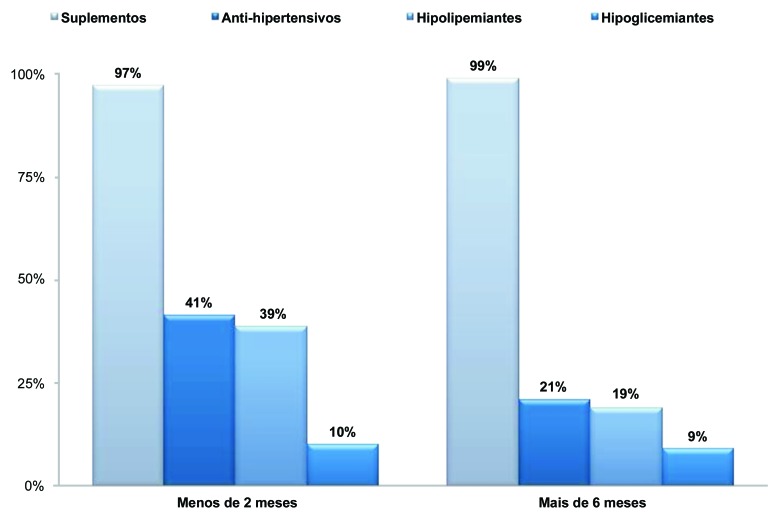
Medicamentos mais utilizados.

Em relação ao perfil farmacoterapêutico de um modo geral, 41% (n = 29) utilizaram algum tipo de anti-hipertensivo para controle da hipertensão arterial, 39% (n = 27) utilizaram algum hipolipemiante e 10% (n = 7) utilizaram hipoglicemiantes orais. Apenas dois pacientes faziam uso de insulina humana antes de 2 meses após a cirurgia. Após 6 meses, apenas 21% (n = 15) utilizavam anti-hipertensivos, 19% (n = 13) utilizavam hipolipemiantes e 9% (n = 6) utilizavam hipoglicemiantes orais, e os dois pacientes continuaram fazendo uso da insulina humana. A Figura 2 mostra as classes de medicamentos mais utilizados pelos pacientes, na qual se destaca o uso de anti-hipertensivos, seguido por hipolipêmicos e hipoglicemiantes.

Quanto ao uso de suplementos, 97% (n = 68) dos pacientes usavam o polivitamínico Centrum® (Wyeth, São Paulo, Brasil) antes de 2 meses. Após 6 meses, 99% (n = 69) passaram a usar o polivitamínico. Quanto à vitamina B12, 69% (n = 48) dos pacientes faziam uso a cada 6 meses, passando para 97% (n = 68) após 6 meses de cirurgia.

## DISCUSSÃO

O estudo mostrou que, dos 70 prontuários analisados, a maioria era de pacientes do gênero feminino. Isso está de acordo com outros estudos nos quais o percentual de mulheres foi predominante. A justificativa provavelmente está no fato de que o gênero feminino se preocupa mais com a estética e a saúde, procurando os tratamentos adequados às doenças, diferentemente do gênero masculino^14^. Observou-se que a faixa etária predominante entre ambos os gêneros variou de 20 a 50 anos, com uma média semelhante à encontrada na literatura^15^^,^ 16.

No presente estudo, a perda de peso foi significativa, uma vez que 97% dos pacientes apresentaram peso abaixo de 105 kg após 6 meses da cirurgia, confirmando os dados reportados anteriormente na literatura. Em um estudo de 2011, foram acompanhados 134 pacientes no período de 8 anos após a cirurgia bariátrica, e observou-se uma significativa diminuição do IMC dos pacientes. O IMC diminuiu de 43,2 ± 4,0 no pré-operatório para 28,7 ± 3,7 no primeiro ano após a cirurgia^17^. Isso está de acordo com o presente estudo, no qual o IMC foi reduzido para 25 kg/m^2^ (23-28) após 6 meses de cirurgia bariátrica.

Um estudo que observou 141 pacientes no período de 6 meses a 4 anos após a cirurgia bariátrica mostrou que os pacientes obtiveram maior redução de peso nos 6 primeiros meses após a cirurgia, quando houve redução de 27%. Conforme os autores, a perda de peso é um dos principais parâmetros para mostrar a eficácia da cirurgia bariátrica, pois, após o emagrecimento, ocorre melhora na saúde do paciente. Isso se deve à baixa ingestão alimentar e ao volume gástrico reduzido, que proporcionam uma melhora significativa ou remissão das comorbidades ocasionadas pela obesidade, como hipertensão arterial, diabetes, problemas cardíacos, entre outros^18^.

Com relação ao perfil metabólico, nossos resultados mostram que os níveis de LDL, colesterol total e triglicerídeos diminuíram gradativamente e que houve um aumento do HDL, fato corroborado por um estudo que acompanhou 130 pacientes antes da cirurgia e até 12 meses depois da sua realização^19^. Uma recente metanálise que englobou 178 estudos mostrou que, após um ano da realização do procedimento cirúrgico, ocorreu uma redução significativa do colesterol total, LDL colesterol e triglicerídeos, enquanto houve aumento do colesterol HDL^20^. Adicionalmente, a redução da glicemia de jejum, observada após 6 meses do procedimento cirúrgico, sugere um potencial papel da cirurgia bariátrica na prevenção e no tratamento da diabetes em indivíduos obesos. A literatura mostra que, após a cirurgia, pacientes mostram redução da hemoglobina glicada e do uso de hipoglicemiantes, o que é associado com a remissão da diabetes. Porém, na prática clínica, muitas vezes os pacientes continuam recebendo medicamentos que não necessitam mais, já que a remissão pode ser subestimada^21^.

Com relação às comorbidades relacionadas à obesidade, entre as doenças apontadas a que mais predominou foi a hipertensão arterial sistêmica. Entre os pacientes, verificou-se que 40% (n = 28) apresentaram alguma comorbidade antes de 2 meses, porém houve redução para 21,4% (n = 15) após 6 meses de cirurgia. Consequentemente, foram reduzidas as dosagens diárias das medicações usadas para controle das doenças associadas à obesidade.

Em um estudo similar que avaliou 130 pacientes, 38 demonstraram uma melhora significativa da hipertensão arterial, reduzindo a dose diária dos medicamentos de controle da pressão arterial. Neste trabalho, verificou-se que o uso de anti-hipertensivos foi retirado de 20 pacientes devido à remissão da doença após 1 ano de cirurgia. Além disso, em 41 pacientes que apresentavam DM2 no pré-operatório, 22 apresentaram uma recuperação completa após 1 ano de cirurgia, e 7 substituíram a insulinoterapia por hipoglicemiantes orais^22^. Outro estudo, que avaliou 88 pacientes, sendo 21 hipertensos antes da realização da cirurgia bariátrica, mostrou que, após 6 meses, apenas dois pacientes apresentavam níveis pressóricos elevados. O mesmo estudo também observou uma redução da utilização de medicamentos anti-hipertensivos^23^. Isso vem ao encontro do presente estudo, pois houve diminuição das comorbidades associadas à obesidade e dos medicamentos após 6 meses de cirurgia bariátrica.

A deficiência de vitaminas e outros nutrientes após a cirurgia bariátrica é observada através do acompanhamento de pacientes no período pós-cirúrgico. A diminuição da ingestão oral de alimentos, bem como a absorção de nutrientes, ocorre devido à restrição ocasionada pela cirurgia. Assim, é indispensável o uso de suplementos após a cirurgia bariátrica^24^^,^ 25. Observamos que, após 6 meses, 99% dos pacientes faziam uso do polivitamínico Centrum® associado à vitamina B12, conforme também demonstrado pela literatura^26^. Essa necessidade de suplementação após a cirurgia pode ser observada no presente estudo, no qual 99% dos pacientes faziam uso de suplementos e 97% faziam uso de vitamina B12 após 6 meses de cirurgia bariátrica.

Além do uso de medicamentos para controle das doenças relacionadas à obesidade, os pacientes utilizavam outras classes, como inibidores da bomba de prótons, antidepressivos, anticoncepcionais orais, medicamentos para tratamento de hipotireoidismo, antiepiléticos, antiasmáticos, analgésicos, anti-inflamatórios, antiplaquetários e sedativos hipnóticos. A classe dos inibidores da bomba de prótons predominou em 58 pacientes da população estudada.

É importante salientar que pacientes obesos apresentam com frequência a função endotelial prejudicada, induzida por uma inflamação crônica de baixo grau, e doença venosa de membros inferiores. Apesar da perda de peso induzida pela cirurgia bariátrica reduzir a quantidade de marcadores inflamatórios, a arquitetura anormal dos vasos pode persistir, e a vasculopatia observada pode estar relacionada a prejuízos em processos de cura de feridas, observados mesmo após a perda de peso^27^. Entretanto, pode ser observada uma melhora em parâmetros de função arterial 6 meses após o procedimento cirúrgico^28^.

Após esgotadas todas as outras terapêuticas, sem êxito por pelo menos 2 anos na redução de peso dos pacientes obesos, a cirurgia bariátrica tem sido de grande eficácia, resultando em uma melhora na qualidade de vida desses pacientes. A cirurgia proporciona a diminuição dos medicamentos de uso contínuo para o controle das doenças ocasionadas pela obesidade, além de melhorar gradativamente o perfil lipídico e os níveis de glicose sanguínea e pressão arterial. Cabe ressaltar a importância do acompanhamento farmacoterapêutico dos pacientes no pré e pós-operatório, já que eles geralmente apresentam problemas de saúde crônicos e utilizam vários medicamentos. Ressalta-se também a importância de um monitoramento por profissionais qualificados no pré e pós-operatório para otimizar os resultados, uma vez que o sucesso do procedimento extrapola o ato cirúrgico em si e envolve uma equipe multiprofissional para a obtenção de melhores resultados de saúde para o paciente^29^.

## CONCLUSÃO

A cirurgia bariátrica promoveu uma melhora significativa das comorbidades, proporcionando um progresso na qualidade de vida dos pacientes e permitindo uma redução na quantidade de medicamentos utilizados.
