# Supplementary material for: Perfil farmacoterapêutico de pacientes obesos no pós-operatório de cirurgia bariátrica
Source: J Vasc Bras. 2016 Jul-Sep;15(3):182–8. [Article in Portuguese] doi: 10.1590/1677-5449.002016 (PMC5829754; doi:10.1590/1677-5449.002016)
Supplement: Translation

# Translation: Pharmacotherapeutic profile of obese patients during the postoperative period after bariatric surgery

**Authors:** Elenara Simoni Kovaleski, Helena Schroeder, Mauricio Krause, Caroline Dani, Patrícia Martins Bock

**Affiliations:** 1 Centro Universitário Metodista IPA, Porto Alegre, RS, Brazil.; 2 Universidade Federal de Ciências da Saúde de Porto Alegre – UFCSPA, Porto Alegre, RS, Brazil.; 3 Universidade Federal do Rio Grande do Sul – UFRGS, Porto Alegre, RS, Brazil.

**Keywords:** obesity, bariatric surgery, attention pharmaceutical, pharmacotherapy

## Abstract

**Background:**

Obesity can be associated with diseases such as diabetes, arterial hypertension and dyslipidemia. Bariatric surgery is one of the most effective treatments available, reducing both weight and comorbidities.

**Objective:**

To evaluate the metabolic and pharmacotherapeutic profile of obese patients after bariatric surgery.

**Methods:**

This is a retrospective, cross-sectional, observational study conducted at a hospital located in the city of Porto Alegre, RS, Brazil, based on analysis of the medical records for 70 patients who underwent bariatric surgery covering periods spanning from 2 months prior to more than 6 months after their bariatric surgery. Statistical analysis was conducted using SPSS 17.0®.

**Results:**

Initial arterial blood pressure was 130/85 mmHg, 6 months after surgery it was 120/80 mmHg (p < 0.01). The metabolic profile 2 months before surgery was as follows: HDL was 34 mg/dL, total cholesterol was 195.07 ± 40.17 mg/dL, LDL was 118.22 ± 41.28 mg/dL, triglycerides were 141.09 ± 43.39 mg/dL, and fasting glycemia was 90 mg/dL. The same figures 6 months after surgery were 43 mg/dL, 133.67 ± 28.14 mg/dL, 65.53 ± 24.3 mg/dL, 104.41 ± 29.6 mg/dL, and 77 mg/dL, respectively (p < 0.01). Use of medications 2 months before surgery was as follows: 41% were on antihypertensives, 39% on hypolipidemics, 10% on oral hypoglycemics, and 97% were on nutritional supplements. These percentages 6 months after surgery had changed to 21%, 19%, 9% and 99%, respectively.

**Conclusions:**

This study illustrates that bariatric surgery for obese patients with comorbidities was successful, demonstrating improvements in their metabolic profile and reductions in use of medications used to treat comorbidities.

## INTRODUCTION

Obesity is a multifactorial, chronic disease that is related to nutritional, genetic, cultural, psychosocial and behavioral factors.^1^ Worldwide, at least 2.8 million people die every year as a result of obesity and its harmful effects. The highest prevalence rates of overweight and obesity are found in the Americas, in both sexes (62% and 26%, respectively).^2^ Obesity is associated with development of many different diseases, such as type 2 Diabetes mellitus (DM2), arterial hypertension, certain types of cancer, and cardiovascular diseases.^3^^,^ 4

Obesity is diagnosed by specialist health professionals who are part of a multidisciplinary team that assesses patients and refers them for the most appropriate treatment. Treatments used to control obesity include medications, low-calorie diets, physical activities, lifestyle changes, cultural changes and, last of all, bariatric surgery. Bariatric surgery is only recommended for people who have morbid obesity or severe obesity associated with comorbidities (body mass index [BMI] ≥ 40 kg/m^2^ or ≥ 35 kg/m^2^ with associated comorbidities).^5^^-^ 7 Bariatric surgery is indicated for patients who have gone through other treatments for a minimum of 2 years, such as pharmacological treatments, physical activity, diet-based treatments, and psychotherapy, and for whom these treatments have not been successful. As such, surgery is indicated as a last resort, but it is one of the treatments that offers greatest efficacy. It consists of surgical intervention involving the stomach or intestine that is intended to reduce the volume of meals eaten and increase satiety signals.^8^

The types of surgical treatment for morbid obesity that have gained acceptance to date are restrictive, malabsorptive, or mixed. The most widely-employed of these is a predominantly restrictive, mixed method called the Roux-en-Y gastric bypass, using the Fobi-Capella technique, which results in consistent weight loss, is well-tolerated by patients, and has an acceptable rate of postoperative complications over the long term.^9^^,^ 10

After this surgery, patients exhibit reductions in the comorbidities that are associated with obesity, such as BMI, bodyweight, arterial blood pressure, glycemia, triglycerides, total cholesterol, and low-density lipoprotein (LDL) and also exhibit increases in high-density lipoproteins (HDL).^11^ However, after the surgery, the patient remains on continuous treatment with antihypertensive medications, hypoglycemics, and hypolipidemics and starts to take supplements.^5^ Administration of supplements, such as vitamin B12, iron, and calcium, among others, is indispensable during the bariatric surgery postoperative period because absorption of these nutrients is reduced, primarily in the intestine, because of the degree of restriction that the surgery causes.^12^^,^ 13

The objective of this study was to evaluate the pharmacotherapeutic profile of obese patients who underwent bariatric surgery at a hospital in South Brazil, at points less than 2 months before and 6 months after the operation, since this profile can change in response to parameters that are altered by the surgery.

## METHODS

This is a retrospective, cross-sectional study, with analysis of qualitative and quantitative epidemiological data. The objective was to evaluate the pharmacotherapeutic profile of obese patients who were taking supplements and medications to treat diseases associated with obesity at a point less than 2 months before and again more than 6 months after bariatric surgery. The study was conducted on the premises of a large hospital in the city of Porto Alegre, RS, Brazil. This is a private hospital that treats patients privately or via health insurance contracts. A total of 70 medical records were analyzed, from patients who were part of a project called the New Weight Group, who met the inclusion criteria, and who underwent bariatric surgery between January 2010 and January 2012.

The medical records selected for the study related to patients who had had bariatric surgery and contained all the information necessary for analysis of pharmacological, clinical, demographic, and anthropometric data. Medical records were rejected if they did not contain all of the information needed for data analysis or if patients underwent surgery, but did not return for follow-up in the New Weight Group.

The variables analyzed were as follows: age; sex; marital status; family history of obesity, diabetes, cardiac problems, arterial hypertension; prevalence of diabetes and arterial hypertension; weight; BMI, waist circumference (WC); arterial blood pressure; laboratory test results (glycemia, triglycerides, total cholesterol and HDL); and all medications used to treat diseases associated with obesity in obese patients within a period spanning from 2 months before to more than 6 months after bariatric surgery.

Data collection was started after the project had been approved by the Research Ethics Committee at the Centro Universitário Metodista IPA, under protocol number 213/2012. Patients’ confidentiality was maintained without negatively impacting them in any way.

The data collected were analyzed using the Statistical Package for the Social Sciences (SPSS) version 17.0®. A descriptive analysis was conducted and the results expressed as frequencies (qualitative variables), means and standard deviations (for variables with normal distribution), or medians and interquartile ranges (quantitative variables). The *t* test was used to compare parameters from 2 months before with those from 6 months after surgery for variables exhibited normal distribution, while the Mann-Whitney test was used for those that did not.

## RESULTS

A total of 70 medical records were analyzed covering a period spanning from 2 months before to more than 6 months after bariatric surgery. Table 1 lists the profile of the sample of patients studied. The distribution of patients’ families’ risk factors was as follows: 75.7% (n = 53) had a history of obesity, 42.9% (n = 30) had a history of diabetes, 54.3% (n = 38) had a history of cardiac problems, and 70% (n = 49) had a history of arterial hypertension.

**Table 1:** Profile of patient sample.

**General characteristics**		
**Variables**	**Absolute frequency (n)**	**Relative frequency (%)**
Sex		
Female	58	82.9
Male	12	17.1
Age		
20-29 years	15	21.4
30-39 years	29	41.4
40-49 years	15	21.4
50-59 years	10	14.3
60-69 years	1	1.4
Marital status		
Single	28	40.0
Married	33	47.1
Divorced	9	12.9
History of obesity		
Yes	53	75.7
No	17	24.3
History of diabetes		
Yes	30	42.9
No	40	57.1
History of cardiac problems		
Yes	38	54.3
No	32	45.7
History of arterial hypertension		
Yes	49	70.0
No	21	30.0

Figure 1 illustrates the frequencies of comorbidities observed in the patients, showing that 47% (n = 33) had arterial hypertension, 12% (n = 9) had DM2, and 7% (n = 5) had cardiac problems at the point 2 months before bariatric surgery, whereas 6 months after surgery, just 36% (n = 25) of the patients had arterial hypertension, 7% (n = 5) had DM2, and 7% (n = 5) had cardiac problems.

**Figure 1:** Diseases associated with obesity.

With relation to this population’s physical activity habits, the study found that just 24% (n = 17) of the sample were engaging in some form of exercise 2 months before surgery and that this proportion had increased to 86% (n = 60) 6 months after surgery. The majority of the population were walking regularly.

Table 2 shows the results for arterial blood pressure, WC, BMI, HDL-C, glycemia, triglycerides, and body weight. Median systolic blood pressure was 130 mmHg (120-150) 2 months before and 120 mmHg (120-130) 6 months after bariatric surgery. Baseline median diastolic blood pressure was 85 mmHg (80-90), falling to 80 mmHg (80-80) after surgery, which is a significant difference in this study (p < 0.01) according to the Mann-Whitney test.

**Table 2:** Metabolic abnormalities.

**Variables**	**2 months before surgery**	**6 months after surgery**
Arterial blood pressure (mmHg)		
Systolic	130 (120-150)	120 (120-130)^*^
Diastolic	85 (80-90)	80 (80-80)^*^
Waist circumference (cm)	127 (118-133)	87 (83-99)^*^
Glycemia	90.50 (86-100)	77.50 (69-84)^*^
BMI	41.44 (39-44)	25 (23-28)^*^
HDL-C	34 (32-40)	43 (41-52)^*^
LDL	118.22 ± 41.28	62.53 ± 24.3^**^
TC	195.07 ± 40.17	133.67 ± 28.14^**^
Triglycerides	141.09 ± 43.39	104.41 ± 29.60^**^
Weight	113 ± 21.5	71.07 ± 14.69^**^

* Values expressed as medians with interquartile ranges and p ˂ 0.01, according to the Mann-Whitney test.

** Values expressed as means and standard deviations with p ˂ 0.01, according to the *t* test.

BMI = body mass index; HDL-C = high density lipoprotein; LDL = low density lipoprotein; CT = total cholesterol.

Median WC was 127 cm (118-133) 2 months before and 87 cm (83-99) 6 months after surgery (p < 0.01), which is a significant difference according to the Mann-Whitney test. Median BMI was 41 kg/m^2^ (39-44) 2 months before and had reduced considerably, to 25 kg/m^2^ (23-28), 6 months after bariatric surgery, which was also statistically significant in this study (p < 0.01) according to the Mann-Whitney test. Mean body weight and standard deviation were 113 ± 21.5 kg 2 months before and 71.07 ± 14.69 kg 6 months after surgery. Two months before bariatric surgery, 59% of the 70 patients had body weight greater than 105 kg, whereas 6 months after surgery, 97% of the patients had body weight below 105 kg and 46% had body weight from 65 to 85 kg (p < 0.01).

Two months before surgery, HDL, total cholesterol and LDL were all at abnormal levels. Median HDL was 34 (32-40), mean total cholesterol and standard deviation were 195.07 ± 40.17, and mean LDL was 118.22 ± 41.28. Six months after surgery, these values had become 43 (41-52), 133.67 ± 28.14, and 65.53 ± 24.3 respectively. Two months before surgery, mean triglycerides and standard deviation were 141.09 ± 43.39, falling to 104.41 ± 29.6 6 months after surgery, which was a significant difference (p < 0.01) according to the *t* test. Median fasting glycemia 2 months before surgery was 90 mg/dL (86-100), falling to 77 mg/dL (69-84) 6 months after bariatric surgery, and these values were also significant (p < 0.01) according to the Mann-Whitney test. At 2 months after surgery, 11 patients, who were not taking medications, exhibited abnormal fasting glycemia results (≥ 100 mg/dL).

With regard to the general pharmacotherapeutic profile, 41% (n = 29) were taking some type of antihypertensives to control arterial hypertension, 39% (n = 27) were taking an hypolipidemic agent, and 10% (n = 7) were taking oral hypoglycemics. Just two patients were taking human insulin up to 2 months after surgery. At 6 months after surgery, just 21% (n = 15) were taking antihypertensives, 19% (n = 13) were on hypolipidemics, 9% (n = 6) were taking oral hypoglycemics, and the same two patients were still taking human insulin. Figure 2 illustrates the drug classes most used by the patients in the sample, with antihypertensive use being most frequent, followed by hypolipidemics and hypoglycemics.

**Figure 2:** Most frequent medications.

The results for supplement use showed that 2 months before surgery 97% (n = 68) of the patients were taking multivitamins (Centrum®, Wyeth, São Paulo, Brazil) and at 6 months after surgery this proportion had risen to 99% (n = 69). Additionally, before surgery 69% (n = 48) were taking vitamin B12 every 6 months, increasing to 97% (n = 68) 6 months after surgery.

## DISCUSSION

This study found that out of 70 medical records analyzed, the majority were from female patients. This is in line with other studies in which the percentages of women were greater than those of men. The explanation probably lies in the fact that women are more concerned about esthetics and health, seeking appropriate treatments for their diseases, unlike men.^14^ It was observed that the predominant age group was in the range from 20 to 50 years in both groups and mean age was similar to reports in the literature.^15^^,^ 16

In the present study, weight loss was significant, since 97% of the patients had achieved body weight below 105 kg 6 months after surgery, confirming data previously reported in the literature. In a study published in 2011, 134 patients were followed for 8 years after bariatric surgery and significant reductions in patients’ BMI were observed. Their BMI fell from a preoperative mean of 43.2 ± 4.0 to 28.7 ± 3.7 during the first year after surgery.^17^ This is similar to what was observed in the present study, in which BMI had fallen to 25 kg/m^2^ (23-28) 6 months after bariatric surgery.

Another study observed 141 patients over a period spanning from 6 months to 4 years after bariatric surgery and showed that patients achieved greatest weight reductions during the first 6 first months after surgery, when the reduction was 27%. According to the study’s authors, weight loss is one of the primary parameters for demonstrating the efficacy of bariatric surgery, since it is after weight loss has taken place that patient health improves, as a result of lower nutritional intake and reduced gastric volume, which provoke significant improvements or even remission of the comorbidities caused by obesity, such as arterial hypertension, diabetes, cardiac problems, and others.^18^

With regard to metabolic profiles, our results showed that levels of LDL, total cholesterol, and triglycerides all reduced gradually, while HDL levels increased, which is in agreement with a study that followed 130 patients from before surgery up to 12 months after it had been performed.^19^ A recent meta-analysis encompassing 178 studies showed that 1 year after the surgical procedure there had been significant reductions in total cholesterol, LDL cholesterol, and triglycerides, while HDL cholesterol had increased.^20^ Additionally, the reduction in fasting glycemia observed 6 months after the surgical procedure suggests that bariatric surgery has a potential role to play in prevention and treatment of diabetes in obese people. The literature shows that after surgery patients exhibit reductions in glycated hemoglobin levels and in use of hypoglycemics, which are associated with remission of diabetes. However, in clinical practice, patients very often continue to take medications that they no longer need, because remission can be underestimated.^21^

With relation to obesity-related comorbidities, systemic arterial hypertension was the most frequent of the diseases listed above that was observed in this sample. It was found that 40% (n = 28) of the patients exhibited some type of comorbidity 2 months before surgery, but this proportion had reduced to 21.4% (n = 15) 6 months after surgery. As a consequence, the daily dosages of medications used to control obesity-related diseases were reduced.

A similar study that assessed 130 patients reported that 38 demonstrated a significant improvement in arterial hypertension and that the daily doses of medications for control of arterial blood pressure were reduced. In that study, 20 patients were taken off antihypertensives in response to remission of their disease 1 year after surgery. Additionally, 22 out of the 41 patients who had had DM2 at the preoperative assessments exhibited full recovery 1 year after surgery and in 7 of them insulin treatment was replaced with oral hypoglycemics.^22^ Another study, assessing 88 patients, 21 of whom had been hypertensive before bariatric surgery was performed, showed that 6 months afterwards just two patients still had elevated blood pressure levels. The same study also observed a reduction in antihypertensive medication use.^23^ This supports what was observed in the present study, since there was a reduction in obesity-related comorbidities and in medication use 6 months after the bariatric surgery.

Deficiencies of vitamins and other nutrients after bariatric surgery is observed during postoperative follow-up of patients. Reductions occur both in oral intake of food and in absorption of nutrients in response to the restriction provoked by the surgery. Supplements are therefore indispensable after bariatric surgery.^24^^,^ 25 We observed that 6 months after surgery 99% of the patients were taking Centrum® multivitamins in combination with vitamin B12, which has also been reported in the literature previously.^26^ This need for supplementation after surgery was observed in the present study, in which 99% of the patients were taking supplements and 97% were taking vitamin B12 6 months after bariatric surgery.

In addition to using medications for control of obesity-related diseases, these patients were also taking other classes of drugs, such as proton pump inhibitors, antidepressants, oral contraceptives, medications to treat hypothyroidism, antiepileptic drugs, antiasthmatic drugs, analgesics, anti-inflammatories, antiplatelet drugs, and hypnotic sedatives. Proton pump inhibitors were the most common, taken by 58 patients in the study sample.

It is important to point out that obese patients often exhibit impaired endothelial function, induced by chronic low level inflammation, and venous disease of the lower limbs. Although the weight loss provoked by bariatric surgery reduces the quantities of inflammatory markers, the abnormal blood vessel architecture may remain, and the vascular disease may be linked with problems with processes involved in wound healing, which may still be observed even after weight loss.^27^ However, improvements in parameters of arterial function can be observed 6 months after the surgical procedure.^28^

When other treatment options have been exhausted, without success for at least 2 years in reducing the weight of obese patients, bariatric surgery has proven of great efficacy, resulting in improved quality of life for these patients. This surgery can lead to reduced administration of continuous use medications for control of the diseases caused by obesity, in addition to gradually improving the lipid profile and blood glucose and arterial blood pressure levels. The importance of pharmacotherapeutic follow-up of patients both before and after surgery is worth emphasizing, since they generally have chronic health problems and take several different medications. Monitoring by qualified professionals before and after surgery is also very important for achieving optimum results, since the success of these procedures is dependent on more than just the surgical operation itself and it requires a multidisciplinary team to achieve the best health outcomes for patients.^29^

## CONCLUSIONS

Bariatric surgery provoked significant improvements in comorbidities, resulting in improved quality of life for patients and allowing the quantities of medications they were taking to be reduced.
